# Recent progress in metasurface-enabled optical waveplates

**DOI:** 10.1515/nanoph-2022-0030

**Published:** 2022-04-01

**Authors:** Yadong Deng, Ziru Cai, Yingtao Ding, Sergey I. Bozhevolnyi, Fei Ding

**Affiliations:** SDU Nano Optics, University of Southern Denmark, Campusvej 55, DK-5230 Odense, Denmark; School of Integrated Circuits and Electronics, Beijing Institute of Technology, Beijing, 100081, P. R. China

**Keywords:** dynamic, multifunctional, optical metasurface, waveplates

## Abstract

The polarization of light is crucial for numerous optical applications ranging from quantum information processing to biomedical sensing due to the fundamental role of polarization as another intrinsic characteristic of optical waves, which is uncorrelated with the amplitude, phase, and frequency. However, conventional optical waveplates that enable polarization control are based on the accumulated retardation between two orthogonally polarized electric fields when light propagates a distance much larger than its wavelength in birefringent materials, resulting in bulky configurations and limited functionalities. Optical metasurfaces, ultrathin arrays of engineered meta-atoms, have attracted increasing attention owing to their unprecedented capabilities of manipulating light with surface-confined configurations and subwavelength spatial resolutions, thereby opening up new possibilities for revolutionizing bulky optical waveplates with ultrathin planar elements that feature compactness, integration compatibility, broadband operation bandwidths, and multiple functionalities. Herein, we review the recent progress in metasurface-enabled optical waveplates, which covers both basic principles and emerging applications. We provide an overview of metasurface-based conventional half- and quarter-waveplates as well as their use in wavefront shaping applications, followed by a discussion of advanced waveplates, including multifunctional waveplates and all-polarization generators. We also discuss dynamic waveplates based on active metasurfaces. Finally, we conclude by providing our outlook in this emerging and fast-growing research field.

## 1 Introduction

Polarization that describes the oscillation direction of electric fields is one of the fundamental and intrinsic properties of optical waves, which is uncorrelated with other properties, such as amplitude, phase, and frequency. Therefore, polarization can contain abundant valuable information and has been widely used in numerous applications, ranging from material property analysis, pharmaceutical ingredient identification, surface topography, remote sensing, to optical communication [1]. However, light emitted from many sources, such as incandescent lamps, consists of an equal mixture of different polarizations, which is referred to as unpolarized light. To produce polarized light with well-defined states of polarization (SoPs) and transform between different SoPs (e.g., linear, circular, or elliptical polarizations), bulky polarization optics have been employed. For instance, optical waveplates made up of birefringent materials (e.g., non-cubic crystals, plastics, and cotton fiber) are used to produce specific retardation between two orthogonally polarized electric fields when light propagates inside the medium. But due to the limited birefringence Δ*n = n*_e_ − *n*_o_ in natural materials (less than 10%), where *n*_e_ and *n*_o_ are the refractive indices along the extraordinary and ordinary axes, the waveplates should be thick enough to provide a sufficient propagation distance much larger than the wavelength [1], going against the growing requirement of miniaturization and dense integration in photonic devices. Besides bulky configurations, conventional waveplates are suffering from limited functionalities and narrow bandwidths. Therefore, it is highly desired to realize miniaturized optical waveplates with excellent performance and diversified functionalities.

Optical metasurfaces, the two-dimensional (2D) analog of metamaterials in the optical range, have attracted increasing attention and rapidly emerged as a promising platform for versatile planar optics due to their unprecedented capabilities of manipulating light with surface-confined configurations and subwavelength spatial resolutions by judiciously engineering the shapes, dimensions, rotations, and locations of planar nanostructures (referred to as meta-atoms) [2], [3], [4], [5], [6], [7], [8], [9], [10], [11], [12], [13]. In particular, among all the fascinating applications, optical metasurfaces open up new possibilities for revolutionizing bulky polarizers and waveplates with ultrathin planar elements that feature compactness, integration compatibility, broadband operation bandwidths, and multiple functionalities. For example, metasurface gratings have been widely implored to demonstrate compact and wideband polarizations [14], [15], [16], [17]. In this paper, we aim to review the recent progress in metasurface-based waveplates (meta-waveplates) over the last few years. Due to the limited contents, hereby we mainly concentrate on the optical range although there are a lot of fancying meta-waveplates demonstrated in the low-frequency ranges [18], [19], [20], [21], [22], [23], [24], [25]. Following the introduction part, we provide an overview of metasurface-based conventional half- and quarter-waveplates (HWPs and QWPs) as well as their use in wavefront shaping applications in Section 2. In Section 3, we discuss advanced meta-waveplates that include multifunctional waveplates and all-polarization generators. Then we summarize the state-of-the-art dynamic meta-waveplates based on active metasurfaces in Section 4. In the final section (Section 5), we provide a summary and outlook for future development in this fast-growing research field.

## 2 Metasurface-based optical HWPs and QWPs

Different from bulky optical waveplates that rely on limited birefringence from the natural materials with non-cubic structures, metasurfaces can provide a strong optical anisotropy at any interested wavelength range by designing nanostructured meta-atoms with distinct and polarization-dependent responses, thereby resulting in miniaturized planner meta-waveplates with excellent and fancy functionalities beyond conventional counterparts. In this section, we start our discussions on metasurface-based HWPs and QWPs.

### 2.1 Metasurface-based conventional HWPs and QWPs

We first consider a typical anisotropic meta-atom with global mirror symmetries, whose main axes are located along *x*- and *y*-directions. From a microscopic perspective, the optical characteristics of this anisotropic meta-atom can be described using the Jones matrix once excited by two linearly polarized (LP) electric fields (e.g., *E*_*x*_ and *E*_*y*_):

(1) $$J_{\text{LP}}=\left(\begin{array}{cc} J_{xx} & 0\\ 0 & J_{yy} \end{array}\right)$$

where $J_{xx}=\left|J_{xx}\right|{\text{e}}^{i\varphi_{xx}}$ and $J_{yy}=\left|J_{yy}\right|{\text{e}}^{i\varphi_{yy}}$ are the transmission/reflection coefficients under *x*- and *y*-polarized excitations, which are mainly determined by the dimensions of the meta-atom along two principal axes. Specifically, $\left|J_{xx}\right|$ and $\left|J_{yy}\right|$ represent the amplitudes, $\varphi_{xx}$ and $\varphi_{yy}$ represent the corresponding phase delays, and $\Delta \varphi =\varphi_{xx}-\varphi_{yy}$ is defined as the relative phase difference. In general, once the amplitudes are equivalent (e.g., $\left|J_{xx}\right|=\left|J_{yy}\right|$ ) and Δφ is equal to ±π/2 or π , meta-QWPs [26], [27], [28], [29], [30], [31], [32], [33], [34], [35] or meta-HWPs [36], [37], [38], [39] could be realized in the optical range. By designing the anisotropic meta-atom supporting detuned resonances, the relative phase difference can be realized in a sub-micrometer thickness, superior to bulky waveplates. Here, it should be emphasized that the recent development of optical meta-waveplates has been largely following the pioneering work on anisotropic metamaterials in the microwave range by Zhou’s group [40].

**Figure 1:**
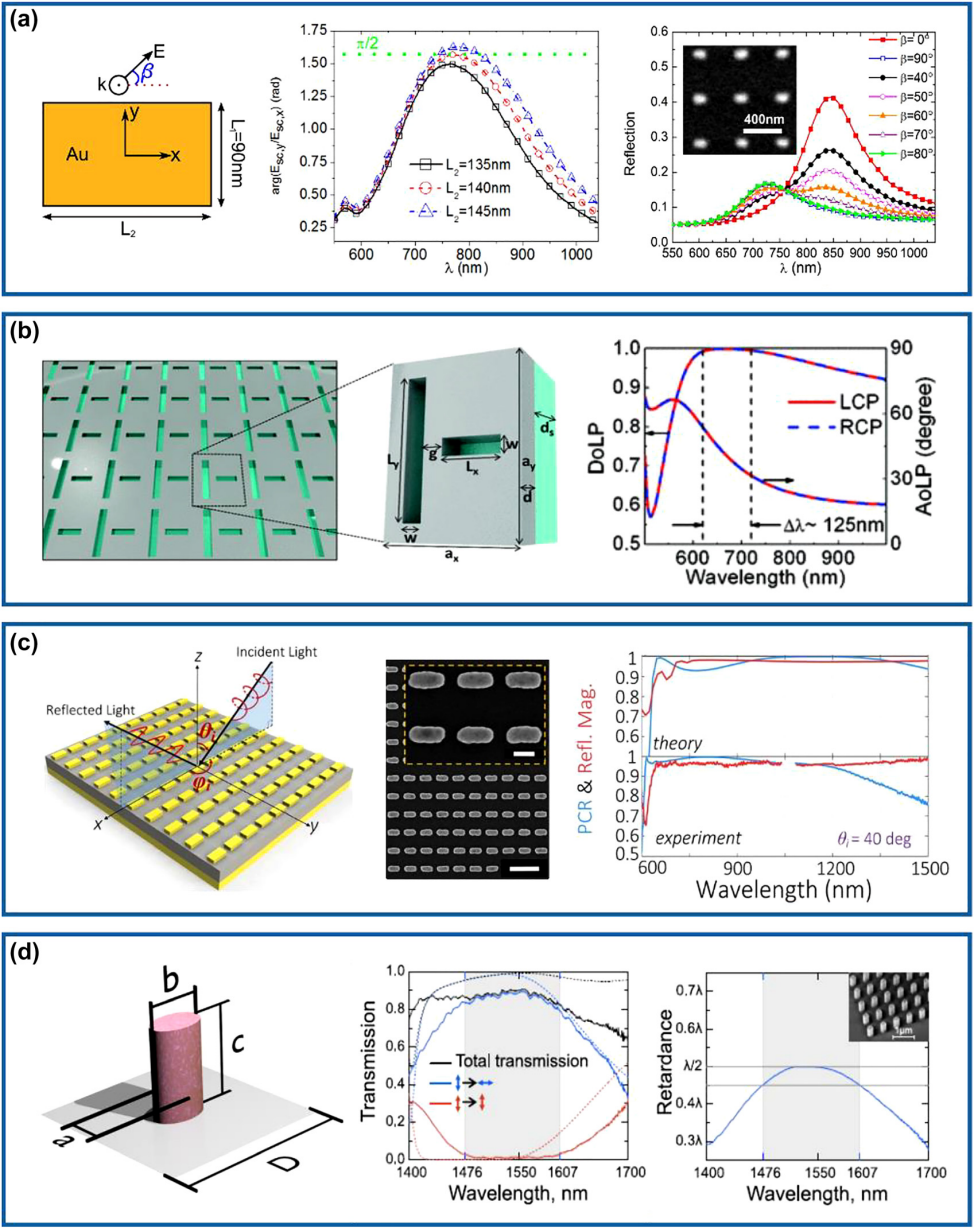
Metasurface-based conventional HWPs and QWPs. (a) Left panel: schematic of an Au brick-shaped meta-atom illuminated with a plane wave with the AoLP of *β*. Middle panel: simulated phase difference of the scattered electric fields for optimized nano-brick structures with different $L_{2}$ . Right panel: normalized reflection spectra for different *β* as a function of the wavelength. The inset shows the scanning electron microscopy (SEM) image of the fabricated Au bricks. Reprinted from Ref. [26]. (b) Left panel: schematic of the complementary plasmonic metasurface with nano slits. Right panel: DoLP and AoLP of the designed nano-slit metasurface for LCP and RCP excited waves as a function of the wavelength. Reprinted from Ref. [27]. (c) Left panel: schematic of the MIM meta-QWP that converts a CP incident wave with an incident angle of ( $\theta_{i},\varphi_{i}$ = 0°) into an LP output wave. Middle panel: SEM image of the fabricated meta-QWP. Right panel: theoretical and experimental PCR and reflection magnitudes as a function of different wavelengths under one incident CP wave with $\theta_{i}$ = 40°. Reprinted from Ref. [33]. (d) Left panel: schematic of the designed pillar-based Si meta-atom as a transmissive waveplate. Middle panel: experimental and theoretical transmission spectra of the designed meta-HWP. Right panel: measured retardance of the fabricated Si meta-HWP as a function of the wavelength. The gray region marks the spectral range with a deviation from λ/2 by less than 10%. The inset shows the SEM image of the fabricated sample. Reprinted from Ref. [39].

Capitalizing on the mechanism introduced above, we would like to discuss some typical examples. Early in 2011, a brick-shaped scatterer supporting perpendicular electrical dipoles was designed by Bozhevolnyi’s group (left panel of Figure 1a), which could generate a phase difference of π/2 in the reflection mode at the center wavelength of 770 nm [26]. By optimizing the dimensions (e.g., $L_{1}$ and $L_{2}$ ) of the gold (Au) nano-brick atop of a substrate, the detuned electric dipole resonances could be supported to realize linear-to-circular polarization conversion under an LP incident beam with different polarization angles. For example, when *L*_1_ = 90 nm and *L*_2_ = 140 nm, the phase difference of π/2 could be satisfied under the LP incident light with a polarization angle of 45° in a narrow bandwidth between 748 and 796 nm (middle and right panels of Figure 1a). Although this work provided a conceptional method for designing subsequent wave retarders, the polarization conversion efficiency of such single-layered waveplates [26, 31, 34, 35] is restricted due to unwanted channels (transmission in this case), and the effective working bandwidth is obviously narrow. Similar to single-layered metallic meta-atoms, their complementary counterparts can be employed to realize optical meta-waveplates [27, 29, 30]. Figure 1b shows a transmissive QWP designed by Zhao et al., where the unit cell is formed by two orthogonal rectangular nano-slits with optimized dimensions in an ultrathin silver (Ag) layer [27]. The phase difference of two LP transmission fields could be maintained to π/2 in a broadband wavelength range from 600 to 800 nm. Within the bandwidth from 616 to 746 nm, the simulated degree of linear polarization (DoLP) approaches 1, and the angle of linear polarization (AoLP) ranges from 28° to 55° with respect to the *x*-axis when the QWP is illuminated with right circularly polarized (RCP) and left circularly polarized (LCP) beams. Similar to single-layered waveplates, this complementary QWP is suffering from limited conversion efficiency due to the unavoidable reflection loss.

To increase efficiency and expand the working bandwidth, gap-surface plasmon (GSP) metasurfaces consisting of an array of metallic meta-atoms, a subwavelength dielectric spacer, and a thick metallic substrate, forming a typically metal–insulator–metal (MIM) resonator [41], have been employed to realize compact optical waveplates [28, 32, 33, 36], [37], [38, 42]. For GSP metasurfaces, the transmission channel is forbidden, and each building block is perfectly reflective. Meanwhile, the designed meta-atom possesses equally high reflection amplitudes and a proper phase difference (e.g., ±π/2 or π ) under linear excitations [43, 44]. For instance, Jiang et al. employed such an MIM configuration to realize the HWP and QWP for highly-efficient and angle-insensitive polarization transformation over more than an octave broad bandwidth that covers the visible and near-infrared ranges [33]. The ultrathin reflective broadband waveplates were designed by optimizing the anisotropic responses of a nanorod resonator array with strong coupling to tailor the interference of light between the top array and the ground plane at a subwavelength scale. As shown in the left and middle panels of Figure 1c, the nanostructure consisting of top Au nanorods, an intermediate silicon dioxide (SiO_2_) layer, and a thick Au back reflector, could reflect a circularly polarized (CP) incident wave into an LP wave. The measured polarization conversion ratio (PCR) is higher than 91% within the expected broadband wavelength range from 640 to 1290 nm for an incident angle up to 40°, and the reflection magnitude could be larger than 92% (right panel of Figure 1c). Although the conversion efficiencies of MIM meta-waveplates could be increased to some degree, Ohmic losses in the metal do exist, especially at the short wavelength region. Therefore, a hybrid MIM metasurface composed of single-crystalline silicon (Si) bricks and an Ag back-reflector separated by a dielectric spacer has been implemented as an efficient HWP in the telecom range [42].

Despite significant progress achieved with MIM meta-waveplates, they are constrained to work in the reflection mode, severely restricting the range of practical applications [45]. As such, all-dielectric anisotropic meta-atoms that consist of high-refractive-index and low-loss materials could also be employed to realize ultrathin optical waveplates [39, 46], [47], [48], [49], [50]. In such all-dielectric metasurfaces, electric and magnetic Mie resonances exploited by corresponding electric and magnetic hotspots with nanoscale volumes could be enhanced with similar strengths at a single frequency or a frequency range, thereby enabling complete and independent manipulations of amplitudes and phases for the transmitted fields [47]. Moreover, transmission efficiencies could be raised hugely since the reflection channel could be suppressed effectively by satisfying the Kerker condition [51]. In addition to the high efficiency, arbitrary and sufficiently large retardation could be generated by designing the optical anisotropy of the meta-atom, which could be much higher compared with the propagation phase accumulated in the birefringent crystals. Therefore, all-dielectric HWPs and QWPs with high conversion efficiencies in the transmission mode have been demonstrated [39, 46, 48]. For example, Kruk’s group designed and experimentally demonstrated a type of transparent broadband all-dielectric metasurfaces composed of closely spaced Si nanopillars to realize high-efficiency polarization manipulation, as shown in the left panel of Figure 1d [39]. According to the generalized Huygens principle, the scattering profiles come from the spectrally overlapped electric and magnetic multipolar modes of the component Si particles, which results in destructive interference in the reflection channel over a broadband range. In terms of the designed meta-HWP (middle panel of Figure 1d), the transmission efficiency is close to 90% and the PCR is near to 99% in the telecom range. In particular, the equivalent birefringence Δ*n* for a *π* phase difference was calculated to be as high as 0.9 at the wavelength of 1550 nm, almost one magnitude larger than those of natural birefringent materials.

At a final comment, it should be mentioned multilayered transmissive metasurfaces that support both electric and magnetic responses and enable constructive interference in the transmission channel can be alternatively used to demonstrate high-efficiency transmissive meta-waveplates [52, 53], which are however mainly limited to the low-frequency ranges due to the intrinsic high loss and fabrication complexity in the optical range.

### 2.2 Metasurface-based multifunctional HWPs and QWPs

In addition to the basic functionality of polarization conversion, advanced wavefront shaping capabilities, such as vortex-beam generation [42, 50, 54], [55], [56], [57], [58], optical holograms [59], [60], [61], [62], [63], [64], [65], [66], [67], [68], [69], [70], [71], [72], and beam steering [73], [74], [75], have realized by spatially integrating multiple HWP and QWP meta-atoms that supply versatile phase modulation.

#### 2.2.1 Multifunctional meta-HWPs using resonance phase

**Figure 2:**
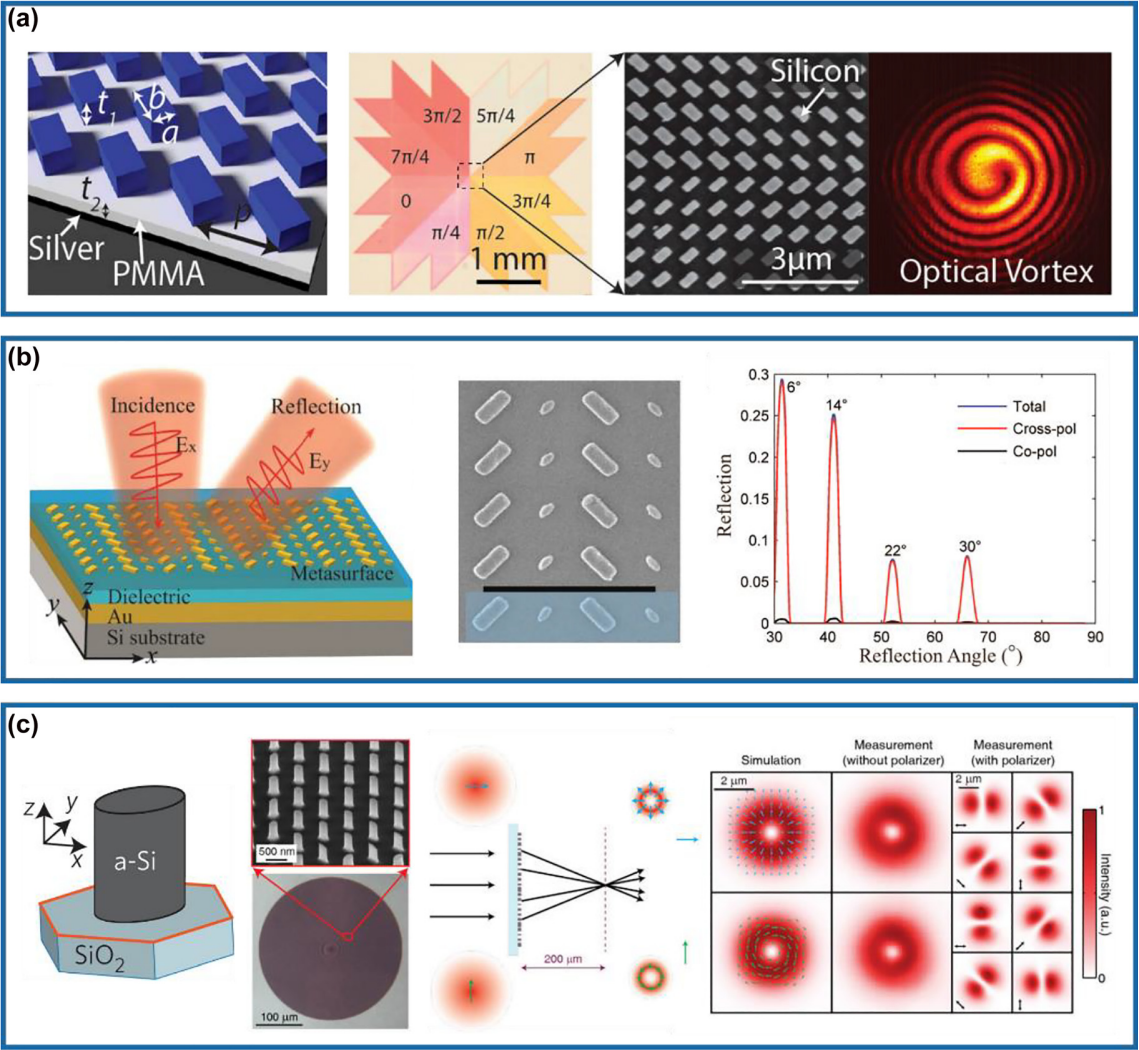
Multifunctional meta-HWPs using resonance phase. (a) Left panel: schematic of a reflective meta-HWP array consisting of Si bricks, a PMMA spacer, and an Ag reflector. Right panel: design and experimental demonstration of optical vortex beam generation in cross-polarization. Reprinted from Ref. [42]. (b) Left panel: schematic of the background-free plasmonic meta-HWP that enables polarization conversion and anomalous reflection in the cross-polarized channel. Middle panel: SEM image of the fabricated structure. Right panel: measured normalized reflection of the fabricated sample as a function of incident angle under *x*-polarized excitation at *λ* = 1000 nm. Reprinted from Ref. [73]. (c) Left panel: schematic, SEM, and optical microscope images of the elliptical Si posts on top of a SiO_2_ substrate. Right panel: illustration and demonstration of focused radially (azimuthally) polarized Bessel–Gauss beam generation under the excitation of an *x*-polarized (*y*-polarized) Gaussian beam. Reprinted from Ref. [46].

The first method towards multifunctional meta-HWPs is to utilize the dimension-dependent resonance phase for the cross-polarized optical fields in a linear polarization basis, which can be realized by designing multiple HWPs enabling efficient linear-polarization conversion along with the complete phase control over cross-polarized fields [42, 54, 73, 76]. As shown in Figure 2a, Yang et al. proposed and experimentally generated optical vortex beams possessing orthogonal linear polarization conversion by employing Si-PMMA-Ag hybrid MIM nano-HWPs to provide an azimuthally dependent spiral phase profile ranging from 0 to 2*π* [42]. By varying geometry dimensions of the topmost Si structures, four different meta-HWPs with relatively high cross-polarized reflectance and phase modulation are selected to provide a phase coverage of *π* for the cross-polarized reflected light with an incremental step of *π*/4. The additional *π*-phase range could be attained by simply rotating these antennas with an angle of 90°, thereby resulting in the full 2*π* coverage with near-unity efficiencies in cross-polarization. The vortex beam could be generated with high efficiency of ∼94.5% in simulation over a wavelength range from 1500 to 1600 nm. In 2015, Ding and co-workers experimentally demonstrated a compact background-free GSP meta-HWP that integrates the functionalities of linear-to-linear polarization conversion and beam-steering in the near-infrared region [73]. As shown in the left and middle panels of Figure 2b, the designed meta-device is composed of four different nano-HWPs in one supercell, which creates a linear phase gradient along the *x*-direction for the cross-polarized reflected fields, leading to the anomalous reflection in the cross-polarized channel [77]. The right panel of Figure 2b shows that the co- and cross-polarized reflected waves could be separately manipulated with the co-polarized reflected wave completely suppressed and the cross-polarized reflected wave dominating in the designed direction, greatly boosting the polarization conversion efficiency. In particular, the cross-polarized reflection power is consistent with the total reflection power and the co-polarized reflection power is extremely low, which effectively proves the required functionality of linear-to-linear polarization conversion and beam steering. In addition, the power rate between the desired cross-polarized field and the orthogonal co-polarized field is above 20, and the integrated PCR is larger than 95%.

Except for plasmonic multifunctional meta-HWPs operating in reflection, all-dielectric meta-HWPs have also been employed to enable multiple functions beyond pure polarization conversion with high conversion efficiencies in the transmission mode [46, 47]. For example, Arbabi et al. designed a Si ellipse-shaped meta-atom with complete control of phase and polarization (the left panel of Figure 2c), through which advanced wavefront shaping could be achieved with high efficiency [46]. As shown in the right panel of Figure 2c, the implemented meta-HWP could convert an *x*-polarized (or *y*-polarized) Gaussian beam into a focused radially (or azimuthally) polarized Bessel–Gauss beam, thereby integrating functionalities of a q-plate and a lens at the same time. Impressively, the measured conversion efficiencies for both *x*- and *y*-polarized waves are higher than 85%, due to the satisfied Kerker condition through spectrally overlapping the electric and magnetic resonances with identical strengths.

#### 2.2.2 Multifunctional meta-HWPs using geometric phase

Whereas aforementioned multifunctional meta-HWPs rely on the resonance phase, with the consequent result that the operating bandwidth is usually limited to the dissimilar dispersion of the different meta-atoms, geometric or Pancharatnam–Berry (PB) phase is dispersionless in the sense that the phase on the cross-polarized CP light is solely determined by the orientation of a single meta-atom [78], [79], [80], [81], [82]. Here, it should be mentioned that the efficiency of the cross-polarized CP channel is still constrained by the dispersion of the meta-atom. But compared to the resonance phase contributed from several meta-atoms with different dimensions and spectral responses, the orientation-induced geometric phase from just one identical meta-atom is more broadband [50, 59], [60], [61], [62], [63], [64], [65], [66, 83], [84], [85], [86], [87]. Here, we take the holograms [59, 64] and metalenses [83, 86] as examples to show the capabilities of multifunctional meta-HWPs.

**Figure 3:**
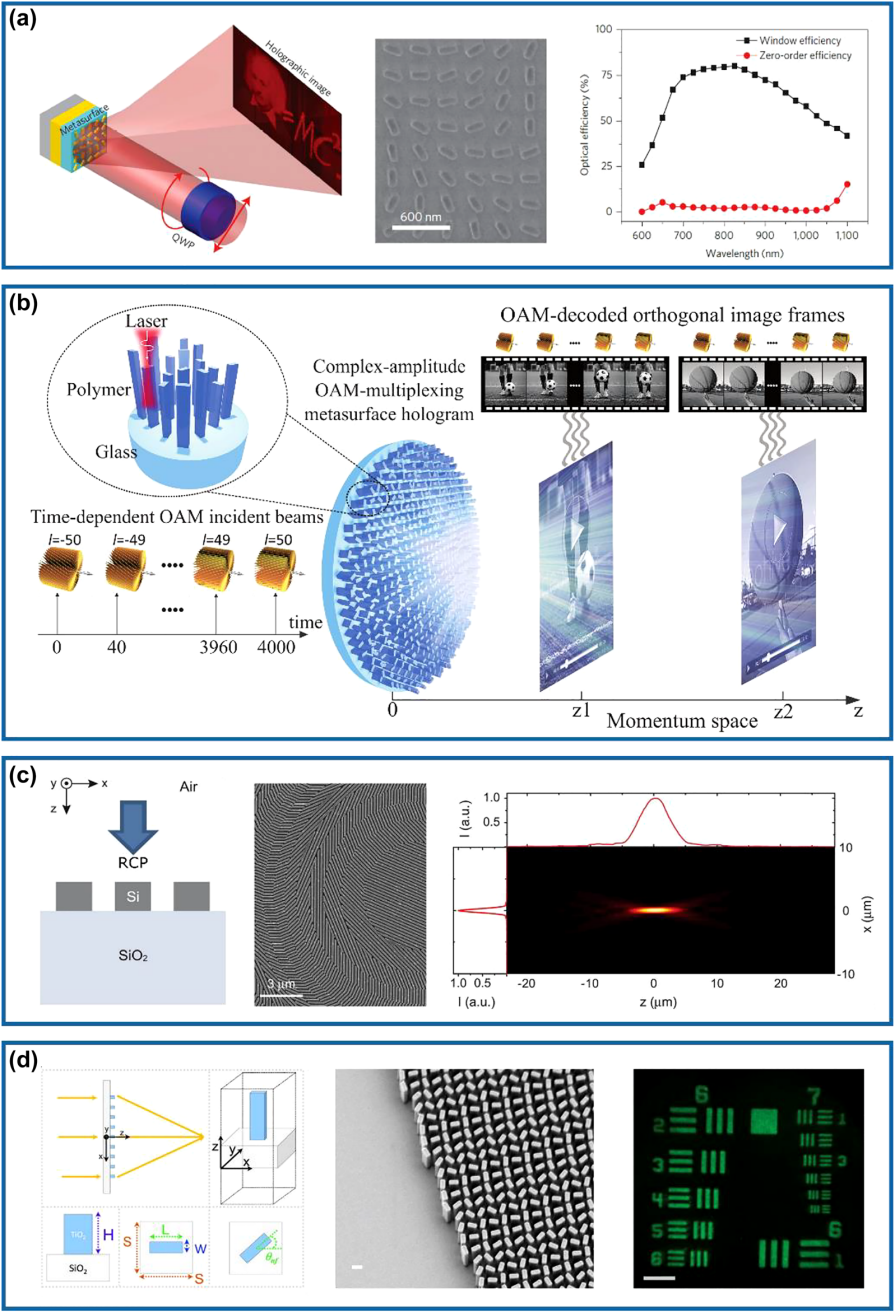
Multifunctional meta-HWPs using geometric phase. (a) Left panel: schematic of the reflective metasurface hologram under a CP incident light. Middle panel: SEM image of part of the fabricated sample. Right panel: measured holographic image and zero-order efficiencies as a function of the wavelength. Reprinted from Ref. [59]. (b) Schematic of the complex-amplitude OAM-multiplexing metasurface hologram that displays two separate holographic videos, both carrying a large amount of OAM-dependent orthogonal image frames, at two different image planes simultaneously in the momentum space. Reprinted from Ref. [64]. (c) Left panel: schematic view of the designed Si meta-HWP. Middle panel: SEM image of part of the fabricated metalens. Right panel: measured intensity distributions of the focusing light passing through the fabricated meta-lens along the vertical and horizontal axes in the *xz*-plane. Reprinted from Ref. [83]. (d) Left panel: illustration of the TiO_2_ metalens and its unit cell. Middle panel: SEM image of the fabricated metalens. Right panel: resolution test image formed by the fabricated metalens at *λ* = 530 nm. Reprinted from Ref. [86].

In 2015, Zhang’s group has realized phase-only geometric metasurface holograms by utilizing spatially-oriented meta-HWPs with diffraction efficiency up to 80% at the wavelength of 825 nm (Figure 3a) [59]. In this design, 16 phase levels have been introduced by precisely tailoring the rotation angle of the designed meta-atoms, which ensures a more robust fabrication quality (middle panel of Figure 3a). Remarkably, the working bandwidth is quite wide, ranging from 630 to 1050 nm with a high measured efficiency greater than 50%. To further increase the fidelity of reconstructed images, a complex-amplitude modulation method can be easily implemented by designing meta-atoms possessing different degrees of circular polarization conversion [64]. As shown in Figure 3b, Ren et al. experimentally demonstrated large-scale complex-amplitude metasurface-based orbital angular momentum (OAM) holography based on three-dimensionally laser-printed polymer nanopillars on the substrate SiO_2_, which enables complete and independent amplitude and phase manipulation. Based on the design, useful information could be extracted by Fourier transform when the incident light carries different OAM modes, thereby reconstructing more than 200 orthogonal image channels encoded with different topological charges and holographic videos without any lenses.

Regarding metalenses based on the geometric phase, a typical example is shown in Figure 3c, where a 100-nm-thick Si gradient metasurface capable of efficiently focusing visible light has been realized from Brongersma’s group [83]. As shown in the left panel of Figure 3c, the subwavelength-spaced Si nano-stripes act as nano-HWPs at the wavelength centered at 550 nm. By discretizing the geometric phase in eight steps, a hyperboloidal phase profile could be approximated to mimic a conventional focusing lens (middle panel of Figure 3c). The fabricated metalens can concentrate the RCP incident light into an LCP focal spot with the focal length and the numerical aperture (NA) of 100 μm and 0.43 at *λ* = 550 nm, respectively (right panel of Figure 3c). Due to the narrow bandgap of Si, the intrinsic loss does exist in the visible spectrum, thereby limiting the efficiency. To increase the efficiency, high-refractive-index, and low-loss dielectric materials with wider bandgaps, such as titanium dioxide (TiO_2_) [86, 88], gallium nitride (GaN) [67, 89], and hafnium oxide (HfO_2_) [90, 91], should be implemented. For example, Capasso’s group has experimentally demonstrated a high-aspect-ratio TiO_2_ metalens with an NA of 0.8, as shown in Figure 3d [86]. The diffraction-limited focusing has been achieved at wavelengths of 405, 532, and 660 nm with corresponding efficiencies of 86, 73, and 66%, respectively. The fabricated metalens could achieve a magnification up to 170× with extremely high image qualities comparable to a state-of-the-art commercial objective.

#### 2.2.3 Multifunctional meta-HWPs using both resonance and geometric phases

Despite significant progress in geometric phase meta-HWPs, their functionalities are limited to the specific CP excitation due to the spin-locked nature of the geometric phase, where the RCP and LCP waves hold the conjugated phases. Currently, this spin-locked limitation has been released by combining the dimension-determined resonance phase and the orientation-dependent geometric phase from multiple meta-HWPs, providing a general scheme for the realization of spin-decoupled functionalities integrated into a single meta-device under orthogonal CP excitations [46, 68], [69], [70], [71, 74, 92], [93], [94], [95], [96], [97], [98], [99], [100].

**Figure 4:**
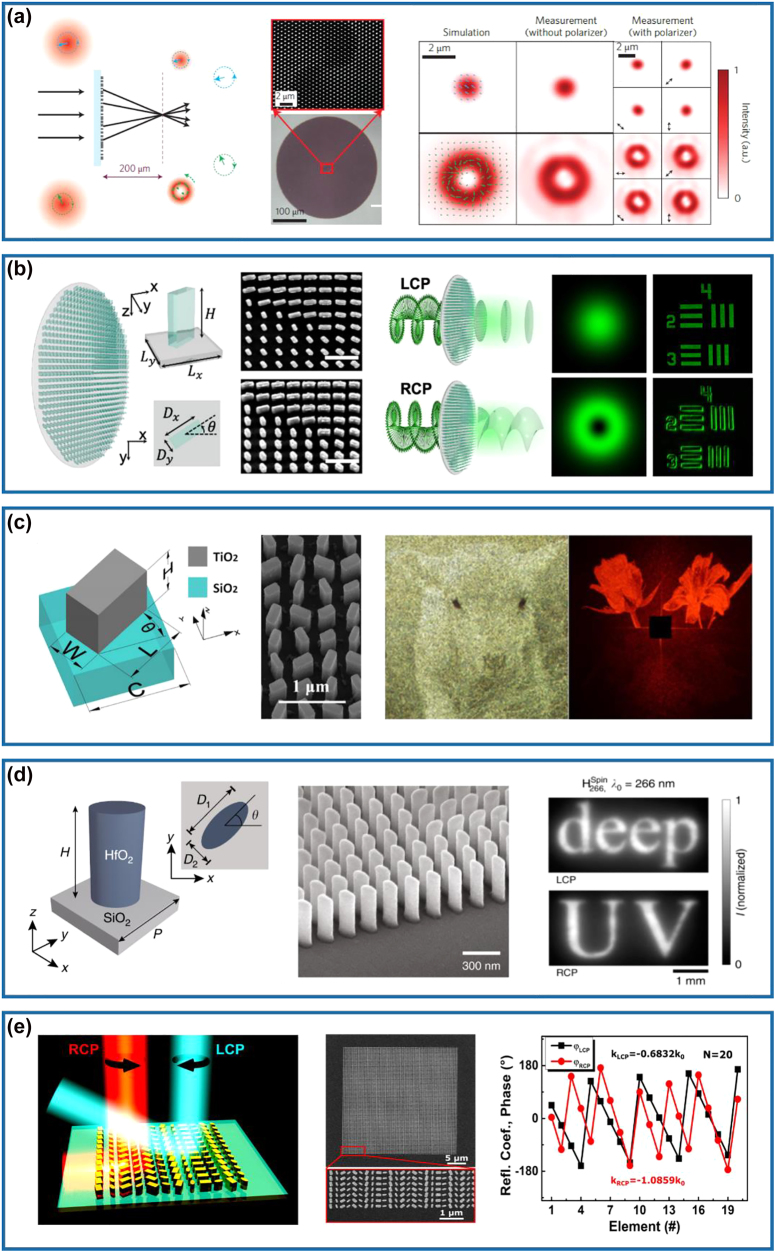
Multifunctional meta-HWPs using both resonance and geometric phases. (a) Left panel: schematic of the Si metasurface that can selectively focus the RCP and LCP light into a nearly diffraction-limited spot and a donut-shaped intensity pattern. Middle panel: SEM and optical microscope images of the fabricated sample. Right panel: simulated and measured intensity profiles. Reprinted from Ref. [46]. (b) Left panel: schematic of the TiO_2_ metasurface and top view of the unit cell. Middle panel: SEM images of the fabricated sample. Right panel: illustration of the spin-selective focused Gaussian and OAM beam generation for bright-field and spiral phase imaging. Reprinted from Ref. [68]. (c) Left panel: schematic of the TiO_2_ unit cell. Middle panel: SEM image of the fabricated sample. Right panel: measured nanoprinting image illuminated with a halogen lamp without filter (left) and measured holographic image with the sample illuminated by LP incident light at 600 nm (right). Reprinted from Ref. [70]. (d) Left panel: schematic of the HfO_2_ unit cell. Middle panel: SEM image of the fabricated sample. Right panel: measured holographic image under LCP (top) and RCP (bottom) incident light at *λ* = 266 nm. Reprinted from Ref. [69]. (e) Left panel: schematic of the spin-decoupled GSP metasurface composed of nano-HWPs with different dimensions and orientations for unidirectional SPP excitation and beam-steering under RCP and LCP incidence, respectively. Middle panel: SEM images of the fabricated structure. Right panel: phase profiles of the reflected fields under RCP and LCP incident light at *λ* = 850 nm. Reprinted from Ref. [74].

In 2015, the first spin-decoupled Si metasurface capable of producing focused OAM beams carrying different topological charges has been realized by Faraon’s group at the near-infrared wavelength of 915 nm [46]. When the RCP and LCP incident beams pass through the designed meta-device possessing a spiral phase profile, the total OAM of the transmitted beams will become *m* = 0 and *m* = 2, respectively. In particular, the RCP incident light is focused into a nearly diffraction-limited spot, while the LCP counterpart is focused into a donut-shaped intensity pattern (Figure 4a). Therefore, the focal spot can be modified rapidly by changing the polarization of the incident light using a phase modulator with a fast response. Based on this concept, Huo et al. experimentally demonstrated a visible spin-multiplexed spatial filter composed of TiO_2_ nano-HWPs, which could realize optical imaging switch between bright-field and spiral phase contrast imaging modes, as shown in Figure 4b [68]. In this design, a constant phase profile is imparted to LCP incident light while a spiral phase profile is imparted to RCP incident light, therefore dynamically realizing switchable ordinary diffraction imaging and isotropic edge detection, respectively. Besides phase modulation, the intensity can be simultaneously encoded within meta-HWPs for nanoprinting [70, 71, 101]. For example, Li and co-workers have proposed and experimentally demonstrated a three-channel metasurface composed of TiO_2_ nano-HWPs, which could generate a near-field nanoprinting image and two far-field holographic images simultaneously, as shown in Figure 4c [70]. In this work, the intensity modulation was governed by Malus’s law and the phase modulation was supplied with both propagation (resonance) and geometric phases. Moreover, owing to the decoupled amplitude and phase algorithm introduced, the proposed multiplexing metasurface could guarantee independence and design freedom in different information channels to the utmost extent. To move the operating wavelength to the ultraviolet (UV) and even deep-UV regimes, Zhang et al. have utilized HfO_2_ to demonstrate multiple high-performance meta-HWPs with resonance and geometric phases (the left and middle panels of Figure 4d) and implemented spin-controlled wavefront shaping functionalities [69]. As shown in the right panel of Figure 4d, a 330-μm-square meta-holographic has been designed to project a holographic “deep” image under LCP incident light and a “UV” image under RCP incident light at the wavelength of down to 266 nm, with the corresponding efficiencies of ∼58.95 and ∼61.23%, respectively.

In addition to the aforementioned spin-decoupled multifunctional meta-HWPs for transmitted optical fields, GSP-based meta-HWPs combining both resonance and geometric phases can be accordingly designed to control the reflected or surface-confined fields. In 2020, Ding’s group has experimentally demonstrated an efficient spin-decoupled multifunctional GSP meta-device that enables unidirectional surface plasmon polariton (SPP) excitation and anomalous beam steering simultaneously under orthogonal CP incident beams in the near-infrared range (the left panel of Figure 4e) [74]. In this work, well-optimized meta-HWPs with different dimensions and orientations that produce two distinct spin-sensitive linear-phase gradients along the *x*-direction are designed. For RCP light, the phase gradient is designed to match the wavevector of SPPs supported by the substrate at normal incidence. For LCP light, the phase gradient is designed smaller than the wavevector of free-space propagating light, which results in an anomalous deflection angle of the reflected light (the middle and right panels of Figure 4e). The fabricated multifunctional meta-device shows efficient unidirectional SPP excitation with a coupling efficiency above 22% in the wavelength range from 850 to 950 nm under the RCP incident light. Once the input is switched to LCP light, the anomalous beam steering with an averaged efficiency of 48% is achieved at the same spectrum.

#### 2.2.4 Metasurface-based multifunctional QWPs

**Figure 5:**
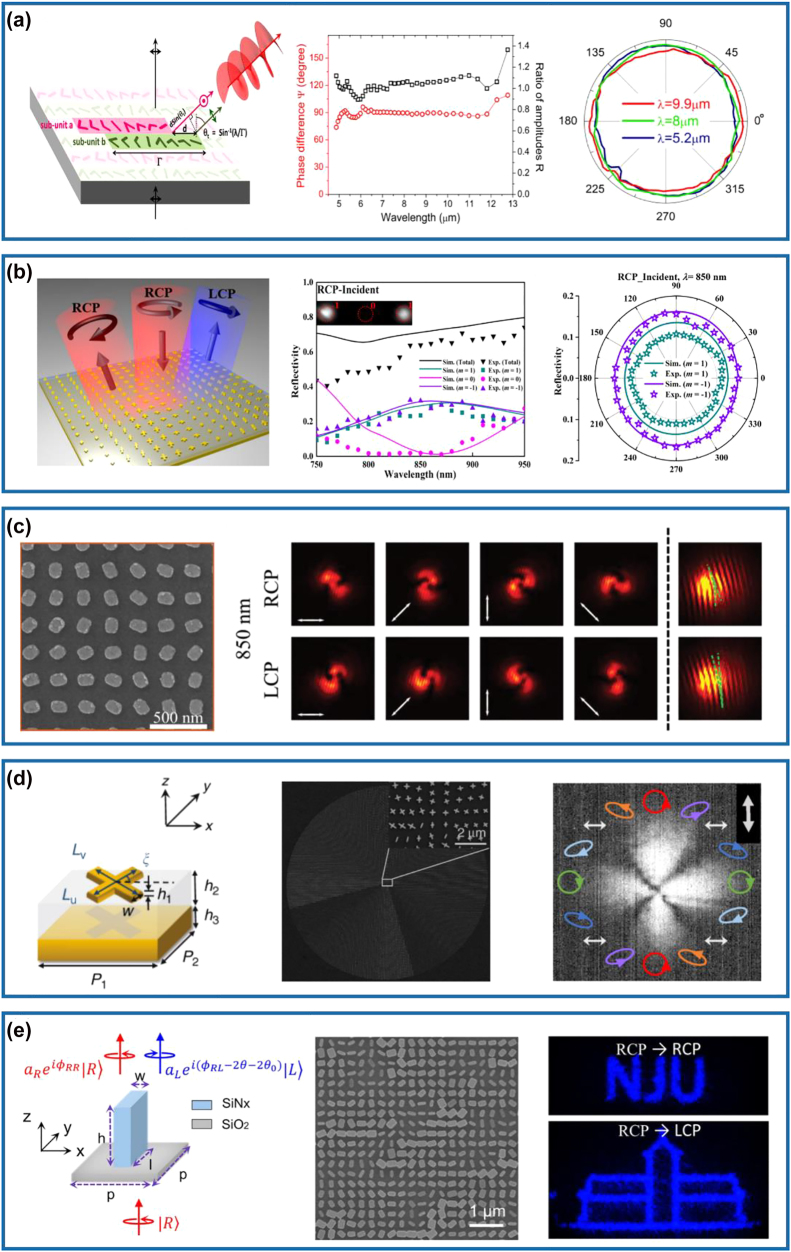
Multifunctional meta-QWPs. (a) Left panel: schematic of the background-free meta-QWP composed of two Au V-shaped antenna subunits. Middle panel: simulated phase difference and ratio of amplitudes between the two scattered waves from the subunits as a function of the wavelength. Right panel: measured SoPs of the extraordinary beam at wavelengths of 5.2, 8, and 9.9 µm. Reprinted from Ref. [102]. (b) Left panel: schematic of the GSP gradient metasurface consisting of four cross-shaped QWP meta-atoms to steer co- and cross-polarized CP waves to −1 and +1 diffraction orders under the RCP incident light. Middle panel: simulated and measured diffraction efficiencies as a function of the wavelengths under the RCP incident light. The inset image shows the measured optical spots of diffraction orders at *λ* = 850 nm. Right panel: simulated and measured SoPs of the steered beams within −1 and +1 diffraction orders under the RCP incident light at *λ* = 850 nm. Reprinted from Ref. [75]. (c) Left panel: the SEM image of the fabricated sample for VVB generation. Right panel: measured intensity distributions of the fabricated sample in the far-field under the RCP and LCP incident light with a linear polarizer at *λ* = 850 nm. The rightmost part shows the interference images of the generated vector vortex beam and a copropagating tilted Gaussian beam. Reprinted from Ref. [56]. (d) Left panel: schematic of the cross-shaped MIM meta-QWP. Middle panel: SEM image of the fabricated structure. Right panel: measured optical image of the generated vectorial optical fields with a linear polarizer tilted by 90°. Reprinted from Ref. [57]. (e) Left panel: schematic of the SiNx nanopillar that acts as a meta-QWP. Middle panel: SEM image of the fabricated structure. Right panel: measured hologram images in the RCP and LCP channels under the RCP excitation. Reprinted from Ref. [72].

The left panel of Figure 5a shows the schematic of a multifunctional meta-QWP that enables simultaneous linear-to-circular polarization and beam steering in an ultra-broadband mid-infrared wavelength range [102]. The designed metasurface composed of two subunits of phased antennas with different dimensions and rotations could generate two transmission waves with consistent propagating directions, equal amplitudes, orthogonal polarization states, and a phase difference of *π*/2 under an LP excitation (middle panel of Figure 5a). Afterward, these two waves will interfere and generate an extraordinary CP wave which is directed in a particular direction away from the ordinary wave, producing a background-free QWP. Impressively, the calculated degree of circular polarization (DoCP) of the steered beam is larger than 0.95 from 6 to 10 μm (right panel of Figure 5a). In addition, the measured suppression ratio between the intensities of RCP and LCP is about 700 at 8 μm, which could prove the excellent performance of the linear-to-circular polarization conversion. However, this meta-QWP is suffering from low efficiency (∼10%) due to the unwanted energy located to the ordinary reflection and refraction channels, which could be solved by using the MIM [32, 33, 36], [37], [38] or all-dielectric [39] QWP designs. For example, relying on two different GSP-based meta-QWPs with high circular-to-linear polarization conversion efficiencies and phase difference of *π* between the reflected LP beams, simultaneous circular-to-linear polarization conversion and power splitting at the center wavelength of 850 nm has been demonstrated by Ding and co-workers [103]. In addition to power splitting, advanced functionalities can be, simultaneously and independently, achieved for co- and cross-polarized waves once more GSP-based meta-QWPs that enable efficient circular-to-linear polarization conversion along with the complete phase control over reflected fields are available [75], superior to multifunctional meta-HWPs that can only use the cross-polarized CP channel. In this design, four cross-shaped meta-atoms acting as nano-QWPs have been designed at the wavelength of 850 nm, with the romance phase covering a wide range up to 300°. By combining both resonance and geometric phases, polarization conversion and beam steering of both co- and cross-polarized fields under the RCP incident light could be realized, as shown in the left panel of Figure 5b. Specifically, upon the RCP excitation, the fabricated metasurface could steer the reflected co- and cross-polarized CP waves into −1 and +1 diffraction orders with the diffraction efficiencies of ∼32 and ∼23.5%, and the measured DoCPs of ∼+90.30% and ∼−92.00%, respectively (middle and right panel of Figure 5b).

Capitalizing on meta-QWPs, it is also possible to produce structured light under CP excitations [55], [56], [57]. The first example is shown in Figure 5c, where vector vortex beams (VVBs) possessing spatially distributed polarization vectors and carrying OAMs have been realized by employing meta-QWPs with azimuthally varied angles [56]. Under the RCP incident light, the AoLP of the reflected LP beam is identical to the azimuthal angle, producing a radially polarized (RP) beam. Meanwhile, this RP beam carries the spiral phase term corresponding to an OAM beam with a topological charge of *l* = −1. Once the incident light is changed to LCP, the output beam will be azimuthally polarized (AP) while the topological charge is reversed (e.g., *l* = +1). Since each QWP element enables broadband circular-to-linear polarization conversion with ∼85% reflection efficiency, the demonstrated VVB generator produces RP and AP OAM beams with measured averaging efficiencies of about 72 and 68% in the wavelength spectrum ranging from 750 to 950 nm for the RCP and LCP incident light. Additionally, the generated VVBs can be considered as a superposition of two CP components: the co-polarized CP component carries no OAM (e.g., *l* = 0) and cross-polarized CP component carrying OAM with a topological charge of *l* = ±2 for the LCP and RCP incident waves, respectively [58, 104]. If more meta-QWPs with different resonances are involved, arbitrary vector optical fields can be implemented [57]. Zhou’s group designed and experimentally demonstrated a vortex beam with varying ellipticity at the working wavelength of 1550 nm, as shown in Figure 5d. In their design, cross-shaped GSP-based QWPs with different resonance phases are properly rotated to supply the additional geometric phase (left and middle panels of Figure 5d). In the experiment shown in the right panel of Figure 5d, four intensity zeros appear when the polarizer is rotated to the vertical direction, manifesting the LP local states. While for the local elliptical or circular polarization states, the intensities with certain strengths are always regardless of the rotation of the polarizer.

Similar to multifunctional meta-HWPs, all-dielectric metasurfaces enable high-performance meta-QWPs with multiple diversified functionalities. Here, we would like to highlight a silicon nitride (SiNx) metasurface QWP with wavefront engineering capabilities for both cross- and co-polarized light simultaneously, as shown in Figure 5e [72]. Based on this platform, an independent spin-sensitive hologram metasurface has been implemented under the RCP incident light, in which the transmitted co-polarized RCP light could be manipulated with the resonance (i.e., propagation) phase to generate a far-field hologram image with “NJU”, while the cross-polarized LCP light could be engineered with both resonance and geometric phases to produce a hologram building image of Nanjing University (right panel of Figure 5e).

## 3 Metasurface-based advanced waveplates

In addition to the aforementioned optical metasurface-based HWPs and QWPs that only perform a single polarization conversion functionality (e.g., linear-to-linear or linear-to-circular polarization conversion), advanced waveplates capable of generating multiple SoPs have been recently developed. In this section, we summarize the advances in this subfield.

### 3.1 Wavelength-multiplexed, direction-multiplexed, and input-polarization-multiplexed multifunctional meta-waveplates

**Figure 6:**
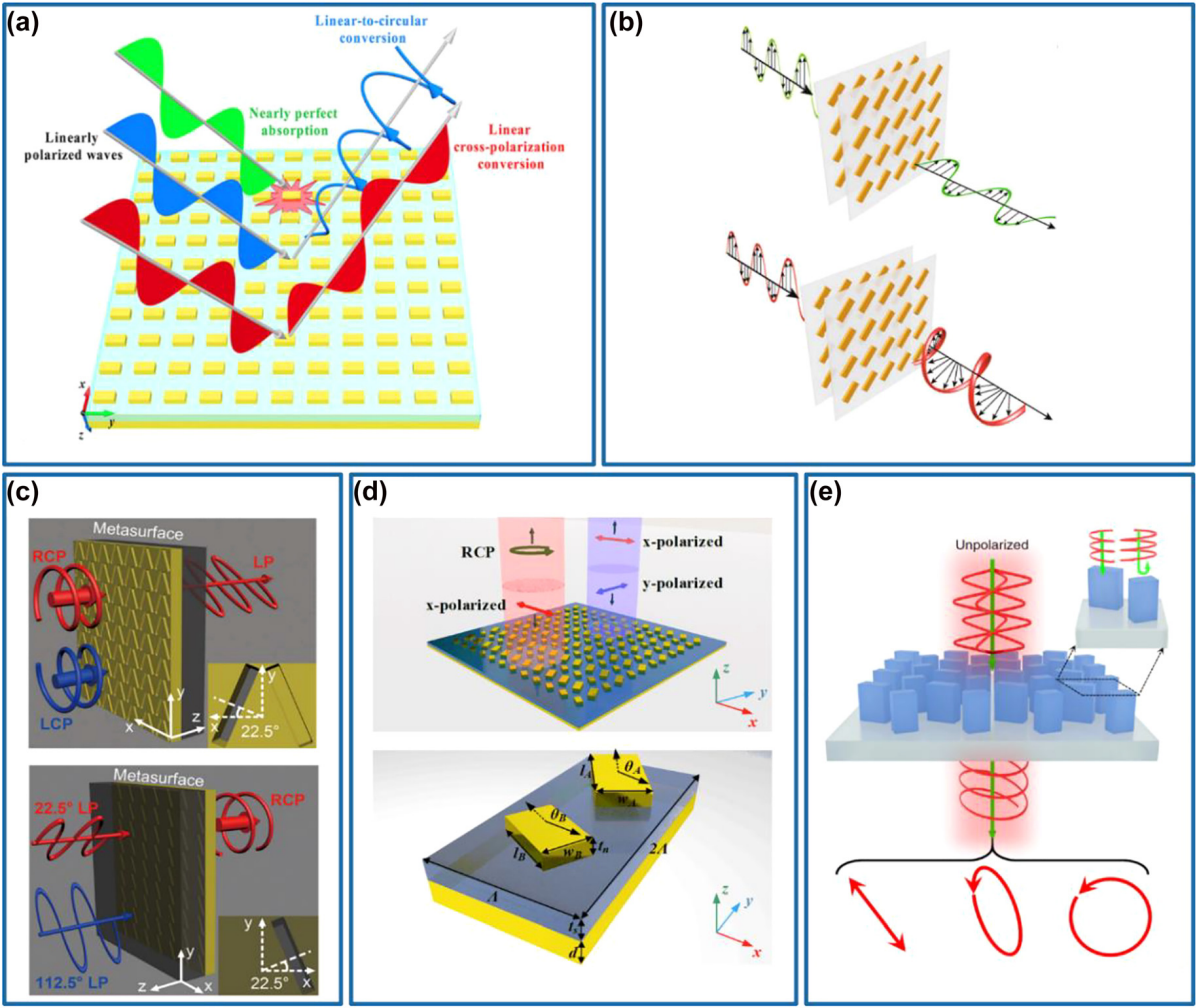
Wavelength-multiplexed, direction-multiplexed, and input-polarization-multiplexed multifunctional meta-waveplates. (a) Schematic of the proposed multifunctional metasurface with the functionalities of nearly perfect absorption, linear-to-circular conversion, and linear cross-polarization conversion at different wavelengths. Reprinted from Ref. [105]. (b) Schematic of the multiwavelength waveplate using a combination of two polarization-sensitive rectangular nanopillar geometries, which functions as an HWP and a QWP at two different and independent wavelengths. Reprinted from Ref. [106]. (c) Top panel: schematic of the metasurface used as a chiral linear polarizer when CP light is incident in the forward direction. Bottom panel: schematic of the metasurface acting as anisotropic circular polarizer when linear polarization light is illuminated in the backward direction. The corresponding unit-cell structures are depicted in the inset with the indicated normal direction of the slits, respectively. Reprinted from Ref. [107]. (d) Top panel: schematic of the dual-functional meta-waveplate that exhibits combined QWP and HWP functionalities for two orthogonal linear polarizations. Bottom panel: schematic of the meta-molecule unit cell. Reprinted from Ref. [108]. (e) Schematic of the all-in-one polarizer that can function at an arbitrary position on the Poincaré sphere, which can directly operate with unpolarized incident light and generate arbitrary polarization states, including linear, elliptical, and circular polarizations, regardless of the incident polarization state. Reprinted from Ref. [109].

Various advanced meta-waveplates possessing diverse polarization conversion functionalities beyond HWPs and QWPs have been accordingly demonstrated by multiplexing the wavelength, direction, or input polarization state [105], [106], [107], [108], [109]. For example, Cheng et al. have theoretically and experimentally demonstrated an ultrathin multifunctional MIM meta-waveplate that has distinct functionalities at different wavelengths [105]. By adjusting the structural parameters of the metal nanorods in the array, nearly perfect absorption, linear-to-circular conversion, and linear cross-polarization conversion have been integrated into one metasurface with high performance across a large range of incident angles (Figure 6a). Similarly, dielectric metasurfaces can be utilized to achieve wavelength-dependent multifunctional meta-waveplates by employing bilayer meta-atoms [106]. As shown in Figure 6b, by independently controlling the geometry and function of each layer composed of rectangular Si nanopillars, Zhou and co-workers have designed a bilayer metasurface that functions as an HWP at a wavelength of 1200 nm and a QWP at a wavelength of 1600 nm. The polarization conversion efficiency of HWP is larger than 80% over the wavelength range from 1180 to 1230 nm while the polarization conversion efficiency of QWP reaches 95% at the wavelength of 1616 nm. Except for multifunctional optical waveplates realized at different wavelengths, multifunctional meta-waveplates that work at a single wavelength also arouse great research enthusiasms. Based on plasmonic stepped slit-groove dimers, a novel direction-controlled bifunctional metasurface polarizer with the operating wavelength of 735 nm has been proposed and experimentally demonstrated by Chen and co-workers, which can be explained by the spin-dependent mode coupling process inside the designed structure [107]. As it is given in Figure 6c, the metasurface behaves as a chiral linear polarizer in the forward direction, which only allows a certain incident spin to transmit and converts it to a specified linear polarization. As for the backward direction, the metasurface acts as an anisotropic circular polarizer, selectively converting a certain linear polarization component into the desired circular polarization. Apart from the direction-controlled bifunctional meta-waveplate, Cai et al. have designed and experimentally demonstrated a reflective dual-functional meta-waveplate based on periodic GSP meta-molecules [108]. In the design, the theoretical derivation was carried out on the meta-molecule assembled of two GSP meta-atoms to obtain the desired reflection coefficients and rotation angles of the two meta-atoms, which meet the requirements of dual-polarization conversion with the *x*- and *y*-polarized incident waves being converted into the RCP and *x*-polarized reflected waves, respectively. Then, two meta-atoms with distinct dimensions and orientations were optimized and selected to comprise the meta-molecule, as shown in the bottom panel of Figure 6d. The fabricated GSP-based waveplate exhibits combined QWP and HWP functionalities with the measured efficiencies of around 73 and 30% at *λ* = 850 nm for orthogonal linear polarizations (top panel of Figure 6d). Furthermore, Wang et al. have presented an effective strategy for designing an all-in-one full Poincaré sphere polarizer with perfect and arbitrary polarization conversion dichroism based on a monolayer all-dielectric metasurface [109]. Based on the matrix transformation of a certain polarization state on the Poincaré sphere, the desired polarization conversion dichroism that allows preferential transmission and conversion of the arbitrary polarization state to its handedness-flipped state while completely blocking its orthogonal state can be physically realized by using two birefringent meta-atoms with distinct dimensions and orientations. The designed arbitrary polarization conversion dichroism metasurface composed of asymmetrically dimerized birefringent crystalline Si meta-atoms exhibits perfect dichroism close to 100% in the simulation and more than 90% in the experiment for arbitrary incident SoPs (Figure 6e).

### 3.2 Angle-multiplexed multifunctional meta-waveplates

**Figure 7:**
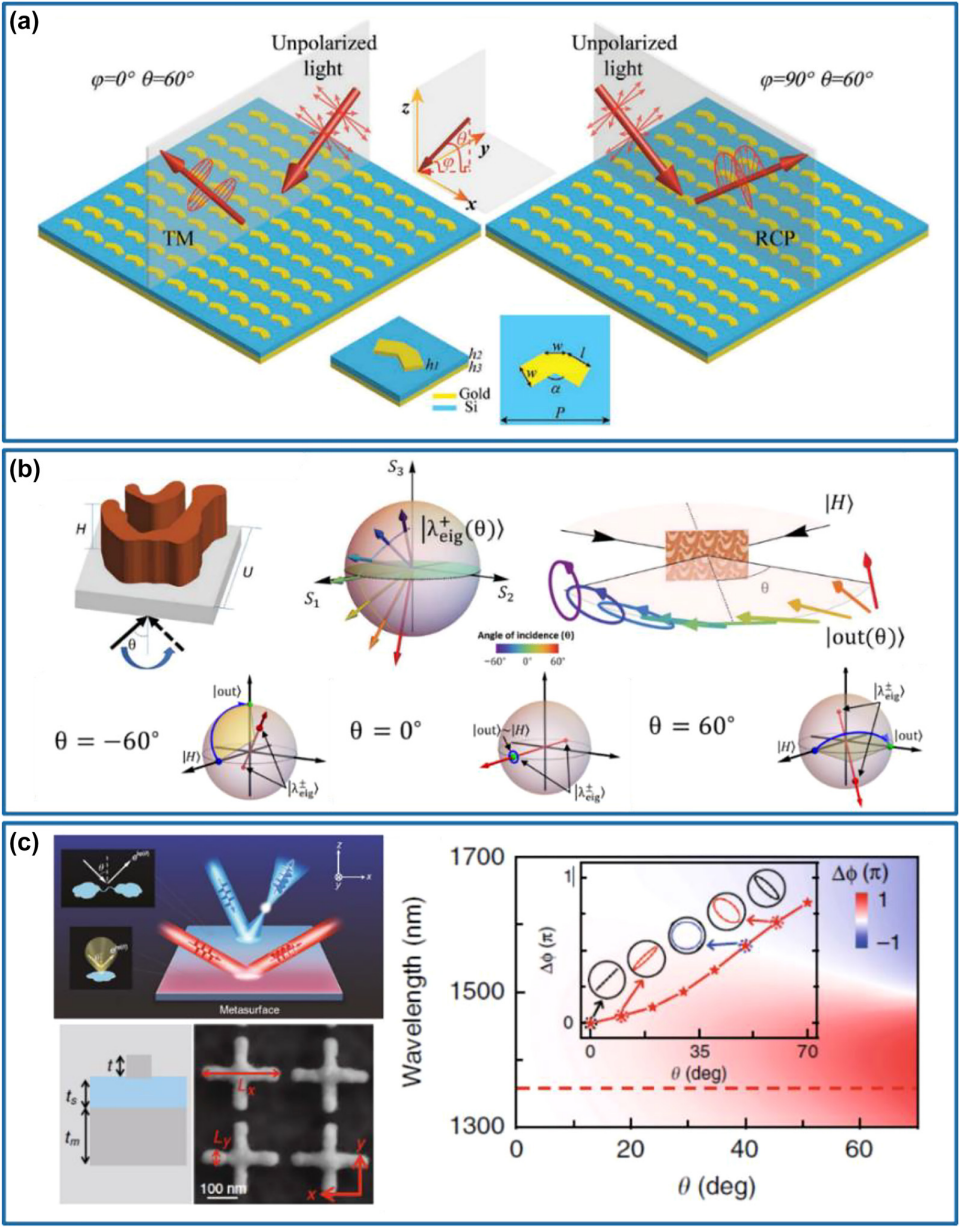
Angle-multiplexed multifunctional meta-waveplates. (a) Schematic of the dual-functional meta-waveplate: for an unpolarized incident wave with 0° azimuthal angle and 60° elevation angle, its TE-polarized component will be totally absorbed and an LP wave with TM polarization can be obtained, while the reflected wave becomes RCP, and the LCP component is absorbed for the incident light with 90° azimuthal angle and 60° elevation angle. The inset shows the 3D and top view of the unit cell. Reprinted from Ref. [110]. (b) Top panel: schematic of the optimized structure and angle-dependent polarization generation. Bottom panel: polarization space representation of the device functionality at different angles. Reprinted from Ref. [111]. (c) Left panel: physical origins of angular dispersions and schematics of the angle-dependent multifunctional meta-device. Right panel: simulated reflection-phase difference between orthogonal linear polarizations versus wavelength and incident angle. The inset shows the reflection phase difference and corresponding polarization states as a function of the incident angle at *λ* = 1358 nm. Reprinted from Ref. [112].

Despite the impressive multiple functionalities achieved for meta-waveplates, most of those polarization-manipulation effects are only demonstrated under normal incidence. Recently, multifunctional meta-waveplates with angle-multiplexed functionalities have also been demonstrated [110], [111], [112]. Huang and co-workers have reported a bifunctional metasurface with the ability to produce 97% linear dichroism and 87% circular dichroism in the infrared region for the LP incident waves with different incident angles [110]. Due to the symmetry breaking of the patterned metal structure, giant linear dichroism and circular dichroism can be realized simultaneously under incident light with orthogonal vertical azimuthal angles, as shown in Figure 7a. However, this design could only generate two different polarization states. To realize more SoPs in the output channel, Shi et al. have demonstrated a topology-optimized dielectric metasurface with continuously transformed birefringence by changing the angle of incidence [111]. In this way, such a single metasurface is able to operate in parallel as different waveplates to achieve different polarization transformations (top panel of Figure 7b). With a varied incident angle, the designed structure is continuously tuned from linear birefringence to elliptical birefringence, thereby enabling versatile polarization transformation. At normal incidence, the eigen-polarization states of the device are LP, which become elliptically polarized at oblique incidence (e.g., −60° and 60°). As a result, for the fixed horizontal linear polarization incidence, the output polarization state changes from the right circular polarization to the horizontal linear polarization and finally to the 45° linear polarization when the angle of incidence varies from −60° to 60° with a step of 60° (bottom panel of Figure 7b). Very recently, Zhou’s group has established a general and systematic strategy to guide the design of optical metasurfaces with fully controlled angular dispersions and experimentally demonstrated an incident-angle-dependent multifunctional waveplate [112]. As shown in Figure 7c, the angular dispersions of the metasurface can be explained by the near-field couplings between meta-atoms and the radiation pattern of a single constituent meta-atom. Based on the derived strategies, they have successfully demonstrated an angle-multiplexed MIM meta-waveplate with a relative phase difference ∆*φ* between two orthogonal linear polarizations varying from 0 to 0.8*π* as the incident angle increases from 0° to 70° at the design wavelength of 1358 nm (right panel of Figure 7c), corresponding to different waveplates operating at off-normal incident angles.

### 3.3 Metasurface-based versatile waveplates for all-polarization generation

**Figure 8:**
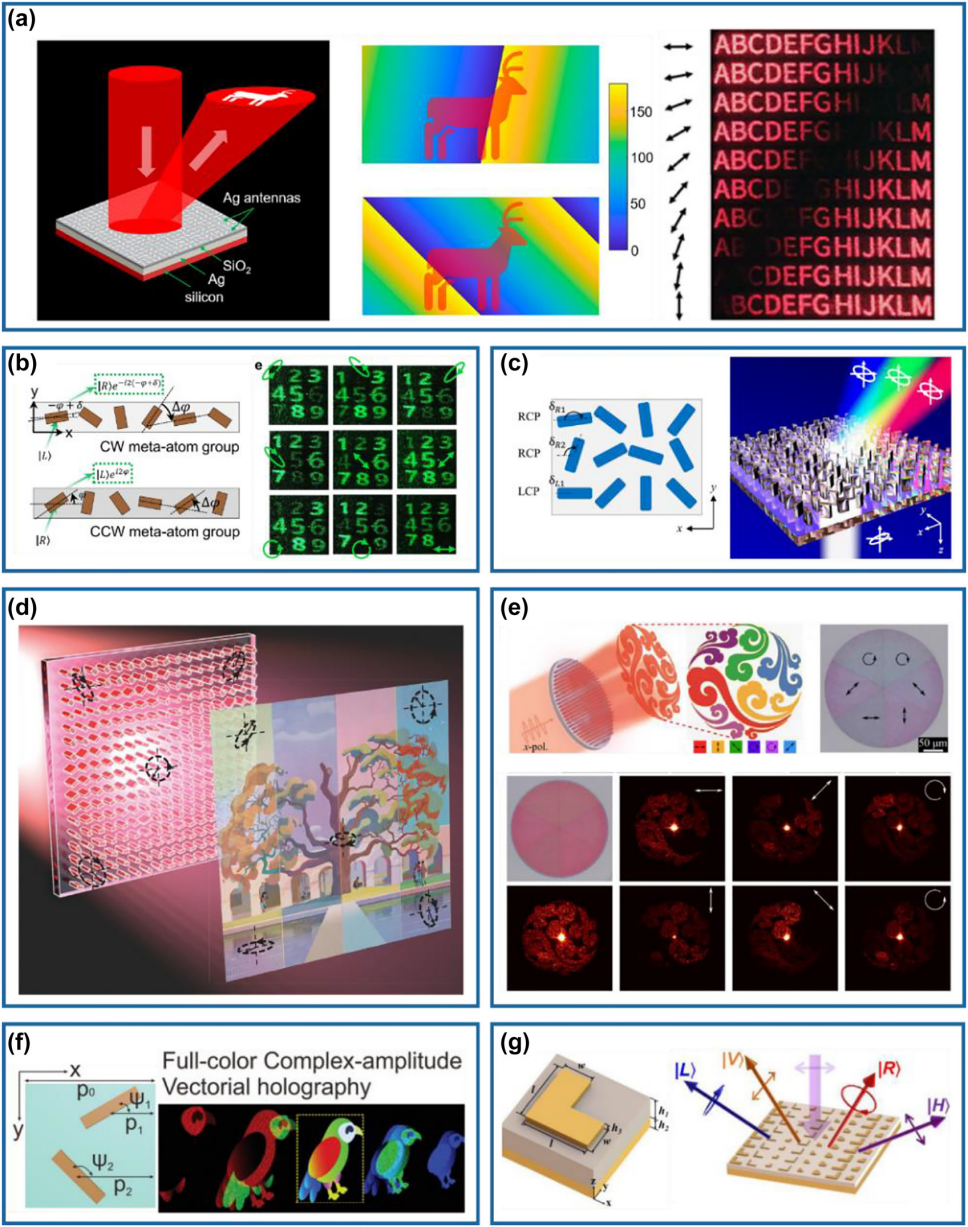
Optical meta-waveplate for all-polarization generation. (a) Left panel: schematic of the metasurface that generates the holographic pattern with continuous polarization distributions. Middle panel: holographic image (deer pattern) with different polarization maps by a slight adjustment of the supercell structure of the metasurface. Right panel: reconstructed holographic images for different polarization directions at *λ* = 635 nm. Reprinted from Ref. [113]. (b) Left panel: two types of meta-atom groups to generate outgoing RCP and LCP with arbitrary phase. Right panel: experimental demonstration of the hologram images carrying nine different polarization states. Reprinted from Ref. [114]. (c) Broadband polarization-maintaining design with a uniform size metasurface. Reprinted from Ref. [115]. (d) Schematic of a metasurface polarization hologram that encodes a polarization pattern into an RGB image. Reprinted from Ref. [116]. (e) Top left panel: schematic of the vectorial holographic display, where the holographic image with spatially-varying polarization states is generated with an *x*-polarized light. Top right panel: optical image of the fabricated meta-hologram. Bottom panel: measured holographic images by illuminating the whole meta-hologram with an *x*-polarized wave at *λ* = 850 nm. Reprinted from Ref. [118]. (f) Left panel: wavelength/angle-independent phase modulations with a diatomic meta-molecule that provides the geometric and detour phases with freely controllable orientations and displacements. Right panel: schematic of the full-color complex-amplitude vectorial meta-hologram. Reprinted from Ref. [120]. (g) Left panel: schematic of the L-shaped MIM meta-atom. Right panel: schematic of the metasurface generating two linear polarization and two circular polarization states. Reprinted from Ref. [122].

Except for the previously demonstrated conventional or multifunctional metasurface-based waveplates, a versatile meta-waveplate that is capable of generating arbitrary and well-defined polarization states and manipulating the corresponding output wavefronts simultaneously has become an emerging research topic. In recent years, some works of metasurfaces-based versatile optical waveplates have been reported with different methods [63, 81, 113], [114], [115], [116], [117], [118]. Tsai’s group has experimentally demonstrated a reflective GSP-based aluminum meta-waveplate to produce six output beams with different SoPs (e.g., four linear polarization and two circular polarization states) in different diffraction channels under an LP incidence [81]. In the design, two supercells composed of spatially orientated HWP meta-atoms proving geometric phase are placed with different offset distances to induce an additional phase difference [80]. Therefore, the anomalously reflected LCP and RCP waves are superposed with needed phase delay to produce the desired linear polarization states. However, the capability of wavefront engineering is limited to beam-steering. Wen and co-workers have used a similar method to realize a vectorial hologram image with a spatially continuous distribution of linear polarization states (Figure 8a) [113]. In this work, a phase-only hologram corresponding to the target image is generated with the Gerchberg–Saxton algorithm and achieved with the geometric phase from metal nanorods. Two types of symmetrically inverted nanorod are interleaved together to form the metasurface, in which two neighboring rows form a supercell. Under the *x*-polarized incidence that contains equal LCP and RCP components and zero phase difference, the obtained holographic image is a superposition of two orthogonal CP images. Based on the displacement of two rows composed of meta-atoms in the supercell of the design, a coordinate-related phase difference between two orthogonal CP images will be generated. As a result, with the determined displacement of two neighboring rows in the supercell, the polarization direction of the combined holographic image varies with the position in the observation plane (middle panel of Figure 8a). As shown in the right panel of Figure 8a, a target image consisting of 13 letters is designed and demonstrated, in which the polarization of the image varies gradually from horizontal polarization to vertical polarization. However, only arbitrary linear polarization states can be achieved with this strategy.

Recently, the geometric phase has been utilized for all-polarization generation and manipulation [63, 114]. For example, Kim and co-workers have proposed a bifunctional metasurface combining both structural color printing and vectorial holography with eight polarization channels simultaneously [114]. The desired bifunctionality is realized from the meta-atom that works as both a Mie-resonator and a waveguide, enabling independent reflection spectra and phase modulation at different locations of the metasurface. Two Si nanopillars are designed to behave as HWPs with the same polarization conversion efficiency but distinct spectral responses. Then, two meta-atom groups composed of meta-atoms rotated in the clockwise and counterclockwise directions with the same angle increment, which allows for the superposition of two orthogonal CP waves with a phase difference of 2*δ* (left panel of Figure 8b) under an LP incidence. Meanwhile, the amplitude ratio between two orthogonal CP components is controlled by the respective numbers of the two meta-atom groups. In this way, the phase and amplitude modulation of the two orthogonal CP output beams can be realized, thereby generating arbitrary polarization states. In order to encode multiple holographic images with different polarization states, a pixelated metasurface is adopted, which consists of nine subpixels that are randomly placed with different meta-atom groups and elimination of unwanted grating effect. The right panel of Figure 8b shows nine holographic images with different SoPs. Due to the utilization of different meta-atoms, the conversion efficiency and propagation phase of the output wave are usually dispersive. As a result, the generation of arbitrary well-defined polarization states may only be implemented at a single wavelength. To address this problem, Song et al. have proposed a general strategy for the full-polarization state generation of diffracted signal over an unlimited wavelength range [115]. As shown in Figure 8c, in order to eliminate the dispersion of the conversion efficiency and propagation phase, only one nanopillar with the uniform size is utilized to compose three geometric phase supercells, where one is arranged in counterclockwise rotated line for the LCP output wave and two are arranged in clockwise rotated line for the RCP output wave. For an *x*-polarized incident wave, the output wave is the superposition of one LCP component and two RCP components, whose polarization state is only related to the starting rotation angle *δ*_L1_, *δ*_R1_, and *δ*_R2_ of the three supercells. Likewise, another configuration with one supercell for RCP output wave and two supercells for LCP output wave is also be obtained in the same way. In this way, arbitrary polarization generation with broadband property can be realized, which is experimentally demonstrated across the entire visible range from 475 to 675 nm. Although the above-mentioned versatile meta-waveplates for all-polarization generation have achieved good results, most of them suffer from the relatively large pixel size and the low efficiency due to two identical beams in two directions and unwanted ghost images generated by the periodicity of the supercells.

Different from the previous demonstrations based on the geometric phase, dielectric meta-atoms working as different types of waveplates with engineered phase responses have been used to efficiently produce arbitrary polarization states with controllable wavefronts and smaller pixel sizes [46, 116], [117], [118]. Faraon’s group has designed a dielectric metasurface platform composed of high-contrast Si elliptical nanoposts to realize complete control of polarization and phase with a subwavelength spatial resolution [46]. Based on such a platform, they have designed a structurally birefringent dielectric metasurface for the realization of vectorial holograms with almost arbitrary polarization patterns for storage and projection of color image data, as shown in Figure 8d [116]. By converting the red-green-blue (RGB) data in arbitrary color images to Stokes parameters, the required field distributions right after the metasurface are obtained. Based on amorphous Si nanoposts, simultaneous and independent control of the phase and polarization of the transmitted wave is achieved on a subwavelength lattice to generate a polarization hologram. Similarly, Ding et al. have adopted Si-based birefringent meta-atoms with engineered birefringence and phase responses for versatile polarization generation and manipulation. By tuning the dimensions and orientations of the meta-atom with rectangular or ellipse cross-sections, the polarization and phase of the transmitted light can be fully and independently controlled with high transmission efficiency at *λ* = 850 nm. Therefore, each meta-atom behaves locally as not only a meta-waveplate but a phase modulator, allowing for the conversion from an LP plane wave to any desired output wavefront with the well-defined polarization state. As shown in Figure 8e, six spatially separated transmitted beams with different polarization states as well as specific wavefronts (i.e., hologram) are simultaneously generated under LP incidence. The corresponding measured efficiency of the fabricated polarization-resolved vectorial hologram is ≈51%.

In addition to the great achievements mentioned above, versatile meta-waveplate for all-polarization generation have also been realized by utilizing diatomic meta-atoms [119, 120] and geometrical-scaling-induced (GSI) phase modulations [121, 122]. For instance, Deng and co-workers have proposed a novel diatomic metasurface consisting of meta-molecules formed by two orthogonally oriented meta-atoms for the reconstruction of holographic images with multiple polarization states [119]. Under oblique incidence, the phase and polarization of the reflected wave are modulated by adjusting the displacements and orientations of two identical meta-atoms. Based on the designed diatomic metasurfaces, they further extended the concept to multi-freedom metasurfaces for full-color complex amplitude vectorial holography (Figure 8f) [120]. By tailoring the displacements and orientations of two identical meta-atoms in each meta-molecule, the combination of the detour and geometric phases are used to simultaneously control the phase, amplitude, and polarization of the impinging wavefront element-by-element. Specifically, the detour and geometric phases are proportional to the displacement between adjacent meta-elements and the orientation angle of the meta-element, respectively, which are completely independent of the wavelength and incident angle. Gao et al. have designed metasurfaces made of L-shaped resonators with different geometrical features to generate different types of polarization states simultaneously, by introducing the GSI phase modulation that depends on the geometrical shape and size of each resonator [121, 122]. Upon illumination, the diffracted waves of each resonator with selected geometrical shape, size, and specific spatial sequence location in the unit cell interacted and led to the generation of multiple beams with different types of polarization states simultaneously. In addition, a matrix inversion approach was utilized to realize an easy, concise, and standard selection process for the unit cell. A specific number of diffracted beams with the desired polarization states are experimentally demonstrated, as shown in Figure 8g.

## 4 Dynamic meta-waveplates

Despite significant advances, the aforementioned optical meta-waveplates are passive and lack real-time tunability, which is not suitable for adaptive photonic integrated systems. In this case, it is crucial to realize dynamic waveplates for active polarization control. Very recently, dynamic optical metasurfaces [123, 124] have been explored to implement dynamic waveplates, where the optical anisotropy can be actively tuned in a wide range by applying external stimuli. In this section, we try to summarize some state-of-the-art dynamic waveplates explored with diverse metasurfaces.

### 4.1 Optically-triggered dynamic meta-waveplates

**Figure 9:**
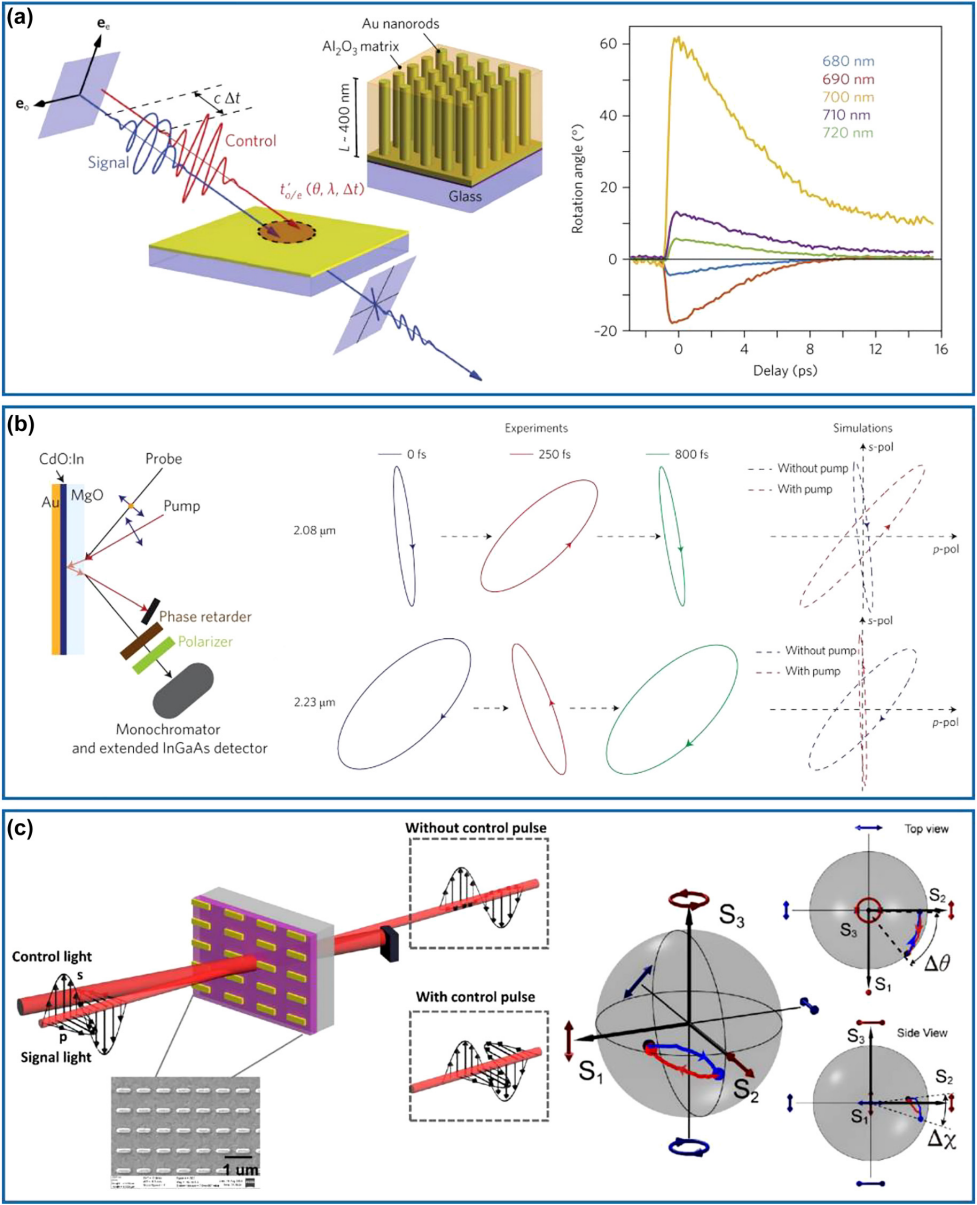
Optically-triggered dynamic meta-waveplates. (a) Left panel: schematic of all-optical switching of visible light polarization with a hyperbolic metamaterial composed of periodic plasmonic Au nanorods in a dielectric matrix, whose anisotropy is first modified by the pump and then probed by time-delayed signal light. Right panel: time-resolved rotation of the polarization ellipse at different wavelengths. Reprinted from Ref. [125]. (b) Left panel: schematic of ultrafast polarization switching with a CdO-based perfect absorber. Middle panel: measured polarization ellipses of the reflected beam at the wavelength of 2.08 and 2.23 μm at a delay time of 0, 250, and 800 fs. Right panel: simulated polarization ellipses of the reflected beam at the wavelength of 2.08 and 2.23 µm with and without a pump. Reprinted from Ref. [126]. (c) Left panel: schematic of the femtosecond light polarization manipulation in an ITO-integrated plasmonic nanoantenna array. Inset shows the SEM image of the fabricated Au nanoantennas. Right panel: dynamics of the polarization state of the transmitted signal light in the Poincaré sphere at the wavelength of 1230 nm. Reprinted from Ref. [130].

To achieve dynamic meta-waveplates, the first method is to optically modify the anisotropy of metasurfaces transiently, thereby resulting in ultrafast switching of light polarization [125], [126], [127], [128], [129], [130]. In 2017, Nicholls and co-workers have demonstrated ultrafast all-optical modulation of visible light polarization in an anisotropic (hyperbolic) metamaterial, which is composed of periodic Au nanorods embedded in a dielectric host matrix using a self-assembly technique, as shown in the left panel of Figure 9a [125]. The assembled hyperbolic metamaterial exhibits a strongly enhanced nonlinear effect near its resonance and results in pronounced changes in the ordinary and extraordinary refractive indices upon the excitation of a femtosecond light pulse. Therefore, the retardation between the transmitted ordinary and extraordinary waves can be optically tuned, leading to a >60° rotation of the polarization state for a signal light pulse at the wavelength of 700 nm with a response time of ∼10 ps, corresponding to a switch rate of 0.3 THz (right panel of Figure 9a). Specifically, this nonlinearity-induced effect has a working bandwidth of around 40 nm near the effective plasma frequency of the designed metamaterial, where different rotation angles can be observed. To decrease the response time to a sub-picosecond level, an ultrafast tunable high-quality factor perfect absorber based on low-loss and high-mobility indium-doped cadmium oxide (CdO) has been proposed and demonstrated at a wavelength of 2.08 μm, close to the epsilon-near-zero (ENZ) wavelength [126]. Upon sub-bandgap optical pumping (left panel of Figure 9b), the ENZ resonance (i.e., Berreman mode) has a pronounced redshift due to the transient increase of the ensembled-averaged effective electron mass of CdO. Therefore, the *p*-polarized reflectance increases from 1.0 to 86.3% while the *s*-polarized reflectance stays the same at the design wavelength, resulting in the polarization switching effect on a timescale of 800 fs for a 45° LP (half *p*- and *s*-polarized) input probe pulse (right panel of Figure 9b). For example, the elliptically polarized reflected wave rotates anticlockwise with the polarization angle rotated from −6° to 47° at a delay time of 250 fs after introducing the pump pulse. Despite the fast response and ease of fabrication, this device has a rather low efficiency since it is working in the perfect absorption regime. Additionally, the dynamic range of birefringence is quite limited. In 2021, Kuidong Wang et al. combined the advantages of the anisotropic nonlinear response of indium tin oxide (ITO) at its ENZ region and polarization-sensitive resonance from anisotropic meta-atoms to achieve a strong and ultrafast modulation of light polarization (left panel of Figure 9c) [130]. For a 45° LP incident signal, this ITO-antenna system functions as a transmissive polarizer without any pump due to the polarization selectivity of the plasmonic antennas and the ENZ material. Specifically, it is transparent for the *s*-polarized component and blocks the *p*-polarized component at the resonant wavelength of 1230 nm. When the femtosecond pump pulse is applied, an anisotropic nonlinear response is induced, leading to a much stronger amplitude modulation along the *p*-polarization direction than that for *s*-polarization one. The device becomes transparent for both *s*- and *p*-polarized light at the original resonant wavelength and the polarization ellipse shows a transient rotation. Compared to Ref. [126] where only the ENZ resonance is optically shifted to a longer wavelength, this coupled ITO-antenna system supplies an additional redshift due to the plasmonic resonance, thereby increasing the anisotropic nonlinearity. As a result, a 32.5° rotation of the light polarization together with a *π*/7 phase change within 600 fs has been demonstrated (right panel of Figure 9c), proving the possibility of a high-speed modulation with a bandwidth of ∼0.73 THz.

### 4.2 Electrically-triggered dynamic meta-waveplates

**Figure 10:**
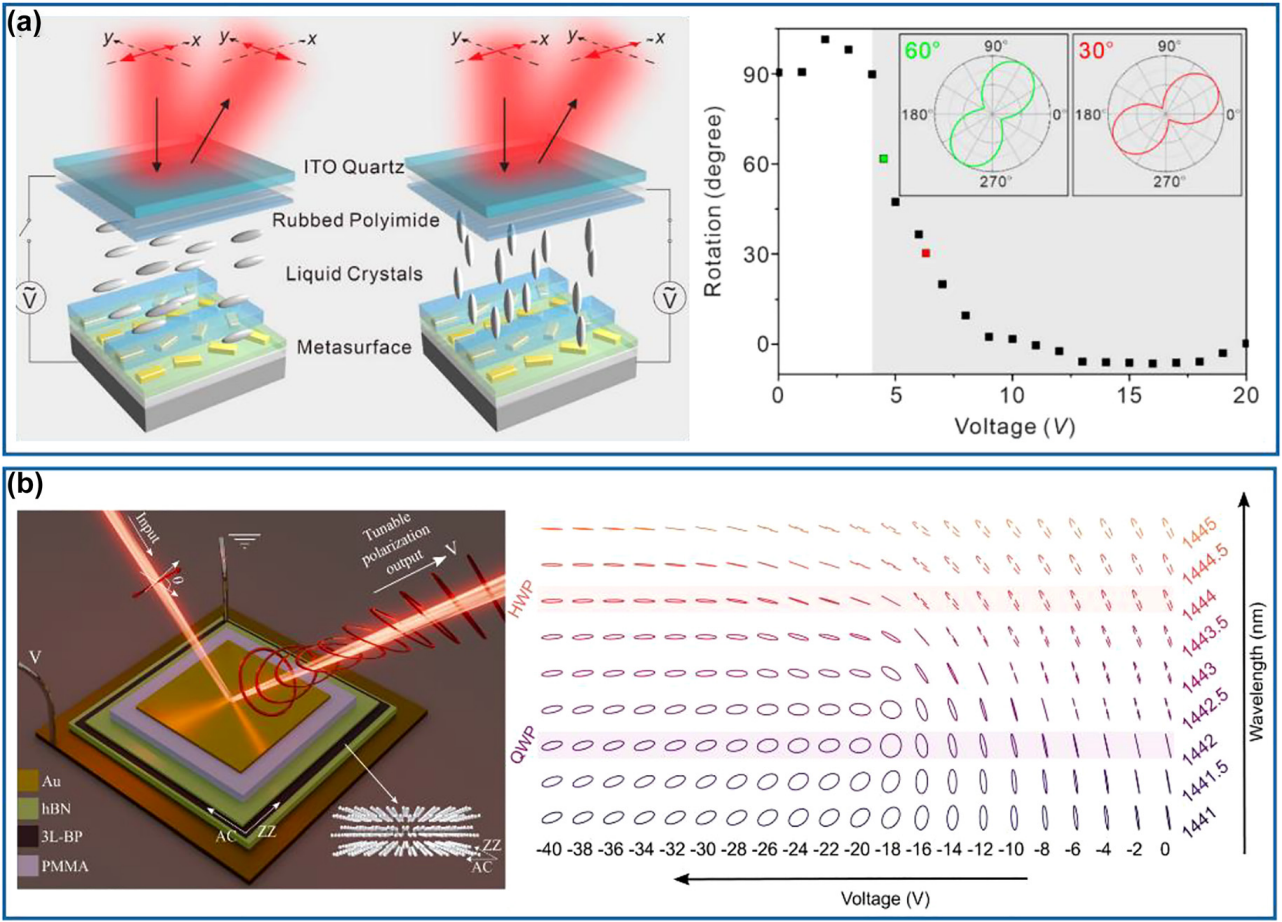
Electrically-triggered dynamic meta-waveplates. (a) Left panel: working principle of the electrically tunable metasurface for active polarization conversion by combining both the geometric from Au antennas and tunable propagation phase supplied by an LC layer. Right panel: measured rotation angle of the linear polarization as a function of the applied voltage. Reprinted from Ref. [132]. (b) Left panel: schematic of electrically tunable polarization conversion with an FP cavity incorporating TLBP with a larger tunability of birefringence. Right panel: measured reflected polarization ellipse for selected voltages and nine different wavelengths. Reprinted from Ref. [133].

In contrast to optically-triggered dynamic meta-waveplates that necessitate ultrafast lasers, electrically tunable meta-waveplates can be stimulated by integrated electronics and thus are naturally appealing for miniaturized reconfigurable photonic networks and systems [131], [132], [133], [134], [135]. Very recently, Liu’s group has experimentally demonstrated a liquid crystal (LC) integrated electrically-driven metasurfaces for polarization conversion at visible frequencies by combing both the geometric phase from Au nanorods and tunable propagation phase controlled by the LC layer of only 410 nm [132], which is different from conventional LC-based devices with µm-scale thickness. As shown in the left panel of Figure 10a, the metasurface supercell consists of two rows of Au nanorods with opposite rotation angles to produce reflected RCP and LCP beams in the same direction upon an LP excitation at normal incidence. By covering these two rows with PMMA and LC layers, the reflected RCP and LCP beams gain different and tunable propagation phases and finally superpose as an LP beam with the rotation angle controlled by the applied voltage. When the applied voltage is increased from 4 to 20 V, the polarization angle can be dynamically tuned from 90° to 0° with a measured switching speed of 100 ms (right panel of Figure 10a). To increase the tunable range of birefringence, tri-layer black phosphorus (TLBP) with electrically tunable optical dichroism has been integrated into a Fabry–Pérot (FP) cavity to realize broadband electro-optic polarization conversion in the telecom range [133]. The reflected beam can be azimuthally rotated or converted to circular polarization for an LP incident beam when voltage is applied between the back reflective mirror and TLBP, as shown in the left panel of Figure 10b. Impressively, the converted SoPs can span nearly half the Poincaré sphere. However, such electro-optic polarization conversion can only work in a narrow bandwidth and the performance is very sensitive to the wavelength. For example, the device acts as a QWP and an HWP at wavelengths of 1442 and 1444 nm, respectively (right panel of Figure 10b). In addition, the efficiency of the reflected beam with modulated polarization state is below 10%. Similar to BP, ITO with tunable carrier concentration has been used to design an MIM metasurface for active polarization manipulation with a smaller birefringence range [134].

## 5 Conclusions and perspectives

In this review, we have summarized the recent advances in metasurface-enabled optical waveplates, ranging from basic principles to emerging applications. Compared with conventional waveplates, meta-waveplates have not only proven to be a unique platform for polarization optics with compact footprints and largely boosted device performance, but also enabled multiple novel functionalities. With the rapid development in this research field, novel concepts and fancy applications have been witnessed almost every day. On the basis of this notion, here we would like to share our perspectives on three promising directions in the future:

(1) Structured light beyond two dimensions. Conventional structured light, such as complex vectorial optical field [57], is often restricted to the two dimensions of the transverse plane, which lacks the capability to control polarization along the propagation direction. As such, the capability of generating 3D structured light beyond two dimensions has received intense interest [136]. Very recently, Capasso’s group has demonstrated polarization transformation along the optical path by combining birefringent metasurfaces and matrix-based holography [137]. Owing to the versatility and feasibility of metasurfaces, 3D structured light with arbitrary spatial distributions of amplitude, phase, and polarization could be implemented in the near future.
(2) Spatiotemporal meta-waveplates. Spatiotemporal metasurfaces that incorporate both spatial- and time-varying modulation of optical fields have become an emerging research field due to the interesting physics and potential applications for ultrafast pulse shaping [11, 138]. Therefore, controlling the temporal polarization state within a single ultrashort pulse could be a fascinating development for truly arbitrary spatiotemporal pulse shaping. We expect high-performance spatiotemporal meta-atoms to appear in this subfield.
(3) On-waveguide meta-waveplates. So far, most of the meta-waveplates can only control the polarization states of free-space light. To meet the increased demand for bandwidth and speed in information processing, photonic integrated circuits (e.g., waveguides) should be able to utilize and manipulate SoPs of guided waves. However, traditional optical waveguides are hindered by limitations of restrained accessible functionalities and bulk footprint. Synergizing metasurfaces with various optical waveguides can largely empower conventional photonic devices [139], [140], [141]. For instance, on-waveguide meta-atoms with a linear phase gradient can bridge the wavevector mismatch between different modes to realize integrated mode converters [140]. However, lots of fundamental and technical issues should be solved before such devices can be really used to arbitrarily generate and manipulate the SoPs of guide modes.
